# Effects of typhoon and upwelling on Chlorophyll-*a* distribution in the northeastern coast of Hainan during Summer

**DOI:** 10.1371/journal.pone.0284689

**Published:** 2023-04-20

**Authors:** Haiyi Shi, Ying Chen, Hui Gao, Hui Zhao

**Affiliations:** 1 College of Chemistry and Environmental Science, Guangdong Ocean University, Zhanjiang, China; 2 Research Center for Coastal Environmental Protection and Ecological Resilience, Guangdong Ocean University, Zhanjiang, China; 3 Southern Marine Science and Engineering Guangdong Laboratory, Zhuhai, China; Guangzhou University, CHINA

## Abstract

Typhoons or upwelling are thought to promote higher phytoplankton chlorophyll-a (Chl-*a*) concentration in many previous studies. However, the combined effects of typhoons and upwelling have been less studied in the South China Sea. Based on satellite remote sensing data, we investigated potential contributions of temperature-characterizing upwelling and typhoon events to Chl-*a* changes in the Hainan northeast area. Results showed that the Chl-*a* concentration was 0.80 mg m^-3^ at the coastal upwelling index (CUI) of 1.7°C in the summer of 2020 when there were no typhoons crossing the area. The CUI (1.01°C) of typhoon-influenced period in 2019 was 0.21°C higher than that of typhoon-free period in 2019. And the Chl-*a* also increased from 0.70 mg m^-3^ to 0.99 mg m^-3^. In comparison, during the typhoon-free period, with the higher CUI, there was the higher concentration of Chl-*a*. In addition, the typhoon affected Chl-*a* concentration is significantly higher than that in the other two typhoon-free periods of 2019 and 2020. Though the typhoon has a limited effect on the upwelling intensity, the Chl-a concentration is much higher than when the upwelling acts alone. This is due to the combined effect of typhoon (vertical mixing and runoff) and upwelling. The above results indicate that upwelling dominated the changes in Chl-*a* concentration in the Hainan northeast upwelling area during the typhoon-free period. In contrast, strong vertical mixing and runoff dominated the changes of Chl-*a* concentration during the typhoon-influenced period in the above area.

## Introduction

**Fig 1:**
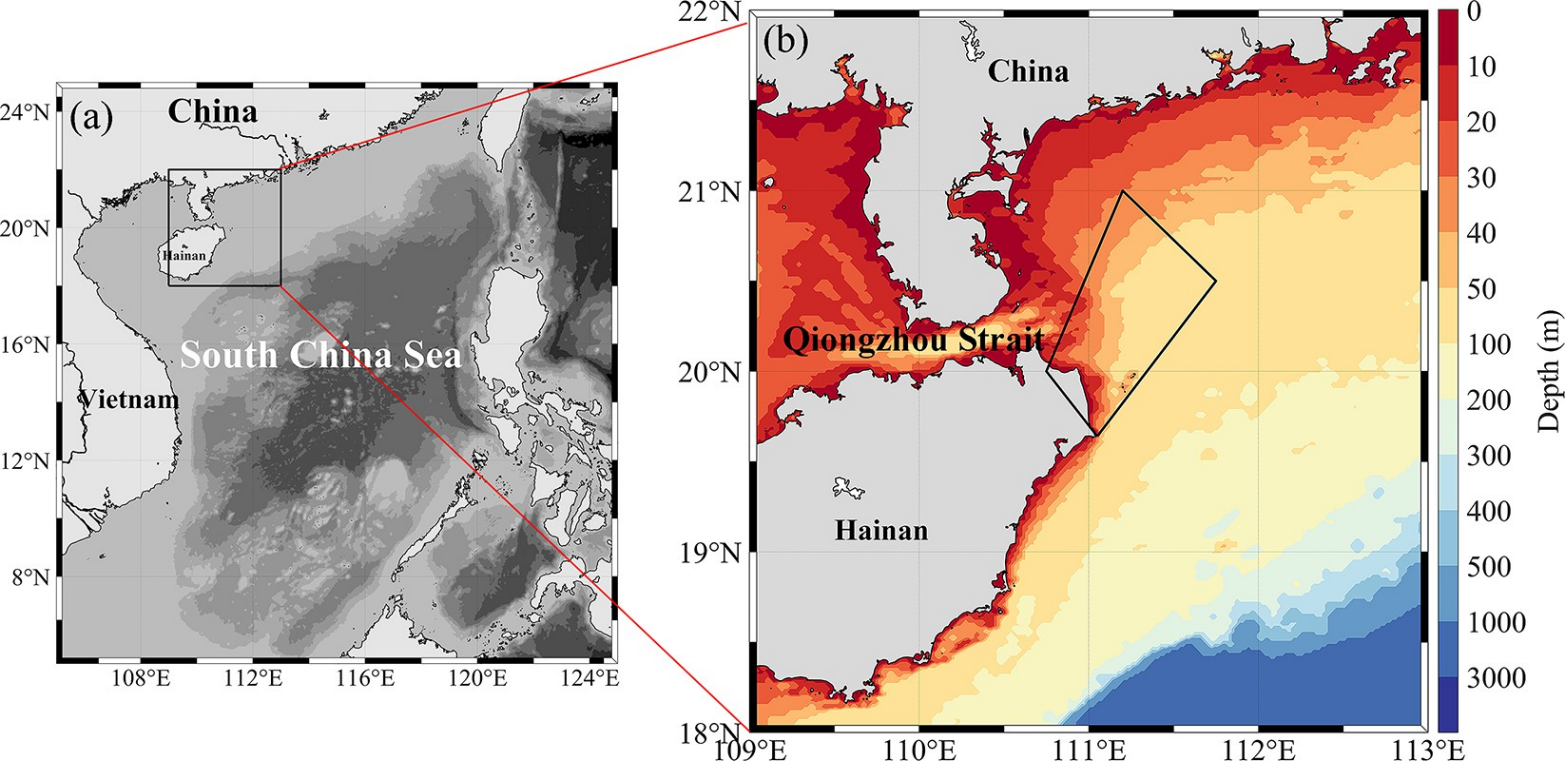
Study area. (a) A map of the South China Sea and (b) deep map of the coastal area of Hainan. Trapezoid in (b) is Hainan Northeast Upwelling (HNEU) area. The color bar is depth with coastal area of Hainan. The high-precision shoreline base-map data used in the map is derived from Global Self-consistent, Hierarchical, High-resolution Geography Database (GSHHG), which is the U.S. Government material and is not subject to copyright protection within the United States.

Upwelling is the vertical upward movement of a water mass that carries cold and high nutrients water from the subsurface layer to the surface layer. And it plays an essential role in phytoplankton primary productivity, fishery production and even the ecosystem of the local area [1]. The South China Sea is a semi-enclosed marginal sea in the northwest Pacific Ocean (Fig 1A). Hainan Island is located in the northwestern part of the South China Sea (Fig 1B). There are multiple upwelling areas along the coast of Hainan Island throughout the summer [2, 3], which complicates the dynamics and biological environment of its adjacent areas [4, 5]. Historical studies show [6] that strong mixing caused by strong winds in winter increases the vertical supply of nutrients inducing phytoplankton growth, resulting in higher phytoplankton chlorophyll-*a* (Chl-*a*) and primary productivity in most areas of the South China Sea. In summer, the stable stratification of the upper ocean leads to the limited nutrient supplement and lower biological productivity in the South China Sea. However, significant Chl-*a* increases are observed in coastal areas of Hainan, especially northeast of Hainan Island, which is mainly contributed by summer upwelling [7].

Phytoplankton Chl-*a* in nearshore is influenced by land-runoff, extreme weather, upwelling, monsoons, and currents [8–10]. Upwelling brings nutrient-rich subsurface and bottom waters to the surface layer, which benefits phytoplankton growth [11] and boosts primary production. The natural upwelling areas account for just 0.1 percent of the world’s oceans, but captures account for more than 40% of total marine fish production [12]. Thus, upwelling plays an essential role in global fisheries production and the global carbon cycle [13].

The coastal upwelling areas of Hainan Island usually has lower seawater temperature and higher phytoplankton Chl-*a* [14]. Upwelling northeast of Hainan is governed by the southeast monsoon along the parallel coast and occurs predominantly in summer (Fig 1B) [2, 15]. Furthermore, the response of the upwelling to the wind direction is different. The southeast wind enhances the upwelling generation in the northern half of Hainan Island, and the southwest wind enhances the upwelling on the eastern side of the island [2]. Typhoons often attack the area near Hainan Island. The "Ekman pumping" formed by typhoon crossing also transports nutrients (particulate nitrogen and phosphorus, etc.) from the deep layers to the surface layer, thus increasing the primary productivity of the surface layer [16]. In addition, the strong near-inertial waves caused by the typhoon can also enhance the vertical mixing in the upwelling area, which makes the temperature and Chl-*a* on the east side of Hainan Island change significantly [17].

It has been shown that the temperature drops of 2–3°C in the coastal upwelling area caused by tropical cyclone is significantly weaker than that in the non-upwelling area (the maximum temperature drops to 7°C in other areas) [18]. When the typhoon crossing the upwelling area, it will have a warming effect on both the upper and lower layers of the ocean [19, 20]. Sometimes the sudden downwelling temporarily suppresses the upwelling, but the upwelling returns after a day. After that, the vertical mixing of the sea is still very strong, which increases the concentration of Chl-*a* [17]. The study found that no typhoons cross only the South China Sea during July 2020 [21]. However, typhoons occasionally crossing the South China Sea in July of other years (1998–2019) (www.typhoon.org.cn). Thus, this study first compares the environmental elements characteristics of Hainan Northeast Upwelling (HNEU) in the summer of 2020 without typhoons to the typhoon-influenced climatology, using satellite remote sensing data. Then, the variabilities of sea surface temperature (SST), wind field (WS) and phytoplankton Chl-*a* concentration in the coastal upwelling zone of northeastern Hainan Island in summer in the presence and absence of typhoon years (months) are explored. Finally, the possible roles of temperature-characterizing upwelling and typhoon events in phytoplankton Chl-*a* variations are revealed in this area. This article would enhance understanding of co-effect of typhoons and upwelling in nearshore areas on phytoplankton, and provide the evidences for further exploring roles of interaction between typhoons and upwelling played in the ecological impacts of typhoons in the upwelling zone.

## Materials and methods

### Study area

The HNEU is located between 19.5–20.5°N and 110.5–111.5°E (Fig 1B). This region is close to the Qiongzhou Strait, has complex topography, and is influenced by both tides and land-runoff, resulting in consistently high phytoplankton biomass throughout the year [1, 22]. This region is mainly controlled by the southwest monsoon in the summer, which is parallel to the coast and carries seawater away from the shoreline to form the Ekman offshore transport. As a result, the region is mostly characterized by wind-generated upwelling [15]. Typhoons frequently occur in summer, characterized by fast-moving speeds and large-scale impact areas [23]. HNEU is one of the most vulnerable areas to typhoons in the South China Sea. Previous study has shown that the occurrence of typhoons will aggravate the vertical disturbance of sea water. It will promote the growth of phytoplankton [24]. We utilized July as the representative month of summer in this study, and monthly averaging of the phytoplankton Chl-*a* data was carried out to evaluate the potential mechanism of typhoon or typhoon-free on phytoplankton Chl-*a* in the HNEU area in summer.

### Satellite products

The merged daily Chl-*a* data from HERMES (https://hermes.acri.fr/) were utilized in this study to examine the spatial distribution characteristics of phytoplankton Chl-*a* in the HNEU area. The data are derived from Sea-Viewing Wide Field-of-View Sensor (SeaWiFS, 1997–2010), Moderate Resolution Imaging Spectroradiometer (MODIS, 2002—present), Medium Resolution Imaging Spectrometer (MERIS, 2002–2012), and Visible Infrared Imaging Radiometer Suite (VIIRS, 2011—present) instruments. The data uses the Garver-Siegel-Maritorena semi-analytical ocean color algorithm [25]. The Chl-*a* data have been widely used to study the distribution characteristics of phytoplankton Chl-*a* in the South China Sea [26]. Considering the large number of clouds in the study area and many gaps in the daily data, this study selects data with a temporal resolution of 8-day averaged and a horizontal resolution of (0.04° × 0.04°) from the merged daily Chl-*a* product.

Sea surface temperature data are provided by the Optimum Interpolation Sea Surface Temperature (OISST) product from the National Centers for Environmental Information (https://www.ncei.noaa.gov/) with a spatial resolution of 0.25° and a temporal resolution of 1 day. The time span from September 1981 to the present. The sea surface wind speed (WS) in our research region was mapped using the Cross-Calibrated Multi-Platform (CCMP) product, which is created by Remote Sensing Systems (https://www.remss.com/) and is accessible every 6 hours with a spatial resolution of (0.25° × 0.25°).

The typhoon data is an online typhoon best track dataset provided by China Typhoon Network (https://www.typhoon.org.cn/) [27], which contains the location and intensity of tropical cyclones in the northwest Pacific (including the South China Sea) every 3 to 6 hours since 1949. It also provides the maximum sustained wind speed and the positions of the typhoon center every 3 hours.

### Upwelling intensity calculation

The Coastal Upwelling Index (CUI) is commonly used to define the intensity of upwelling [28]. Ru Zhang applied a modified CUI to quantify the wind-generated upwelling in the Chinese offshore [29]. The coastal upwelling index is usually calculated by using the temperature difference between the center of upwelling and the outer sea at the same latitude [30].


$$CUI={SST}_{offshore}-{SST}_{coast}$$ (1)

Where *SST*_*offshore*_ is the sea temperature in the non-upwelling area at the same latitude as the upwelling center, and *SST*_*coast*_ is the temperature of the upwelling center. The difference between the two is taken to characterize the intensity of the upwelling. In this study, the area of 400 nautical miles from the upwelling area at the same latitude is defined as the non-upwelling area.

### Pearson correlation coefficient

Pearson correlation is a statistical method used to evaluate the linear relationship between two variables [31]. In this study, we use Pearson correlation to examine the relationship between two variables, which helps us understand their association. The Pearson correlation coefficient is calculated by computing the covariance between two variables, and then dividing it by the product of their respective standard deviations. This produces a value between -1 and 1, indicating the degree of correlation between the two variables. A positive Pearson correlation coefficient indicates a positive correlation between the two variables, while a negative coefficient indicates a negative correlation. The larger the absolute value of the Pearson correlation coefficient, the stronger the correlation. Additionally, a significance level can be used to determine whether the correlation coefficient is statistically significant. In this study, we will use a significance level of *p* < 0.05 to indicate statistical significance of the correlation coefficient [32].

## Results

### Spatial distribution of SST and WS

**Fig 2:**
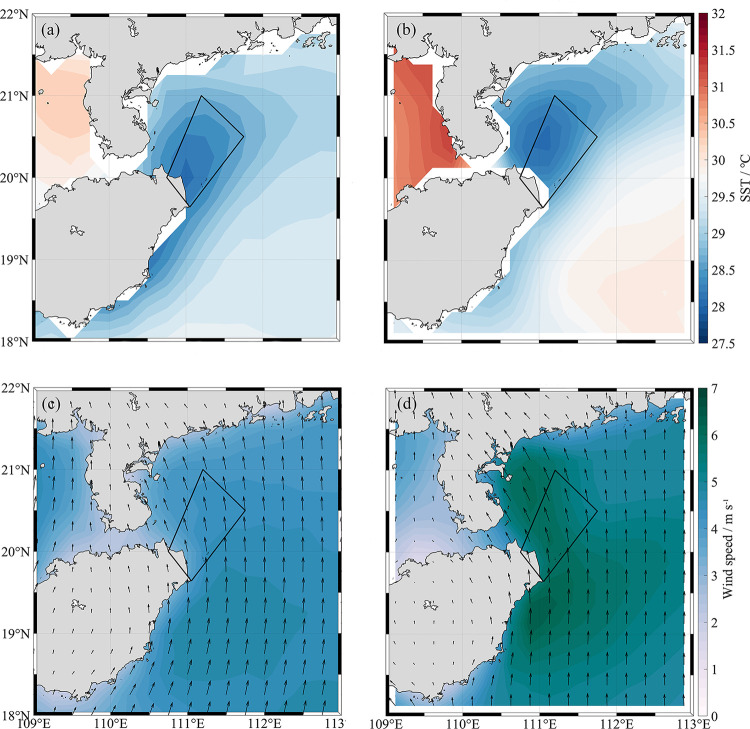
**Temperature and wind fields in July.** Monthly climatology of (a) sea surface temperature (SST) and (c) wind speed (WS) averaged in July from 1998 to 2019, and (b) the summer (July) mean SST and (d) WS in 2020. The high-precision shoreline base-map data used in the map is derived from Global Self-consistent, Hierarchical, High-resolution Geography Database (GSHHG), which is the U.S. Government material and is not subject to copyright protection within the United States.

Fig 2 shows the spatial distribution of SST and WS climatologies. And SST and WS in July 2020 (without typhoon) in the northeast of Hainan Island. Fig 2A and 2B show that the SST in the northwest of Hainan Island is higher than that of the southeast side by about 1°C. A low temperature center (trapezoidal area in Fig 2A and 2B) exists along the northeast coast of Hainan Island with temperature ranging from 28°C to 28.5°C. An increasing temperature gradient from the center to the surrounding area occurs. The above features suggest the existence of local upwelling in this area. Compared with the climatological SST, the SST in the summer of 2020 is slightly higher. But the central temperature of HNEU is lower than that of climatology. And the temperature gradient between the low-temperature center and the surrounding sea in 2020 is significantly larger than the climatology. In addition, the location of the upwelling shifts significantly northward compared to the climatology, indicating that an upwelling area formed closer to the east coast of the Leizhou Peninsula. The study area of both climatology and 2020 are affected by south or southwest winds in summer (Fig 2C and 2D). However, affected by the topography of Hainan Island, the local wind direction in HNEU area changes to southeast wind. The climatological WS was found to be between 4 m s^-1^–6 m s^-1^ (Fig 2C). The overall WS of the study area shows a decreasing trend from south to north. WS around HNEU is roughly 4.5 m s^-1^. While the WS in 2020 ranges from 5 m s^-1^ to 7 m s^-1^, the wind speed near the HNEU is high, roughly at 6.5 m s^-1^.

### Spatial distribution of Chl-*a* concentration with and without typhoon

**Fig 3:**
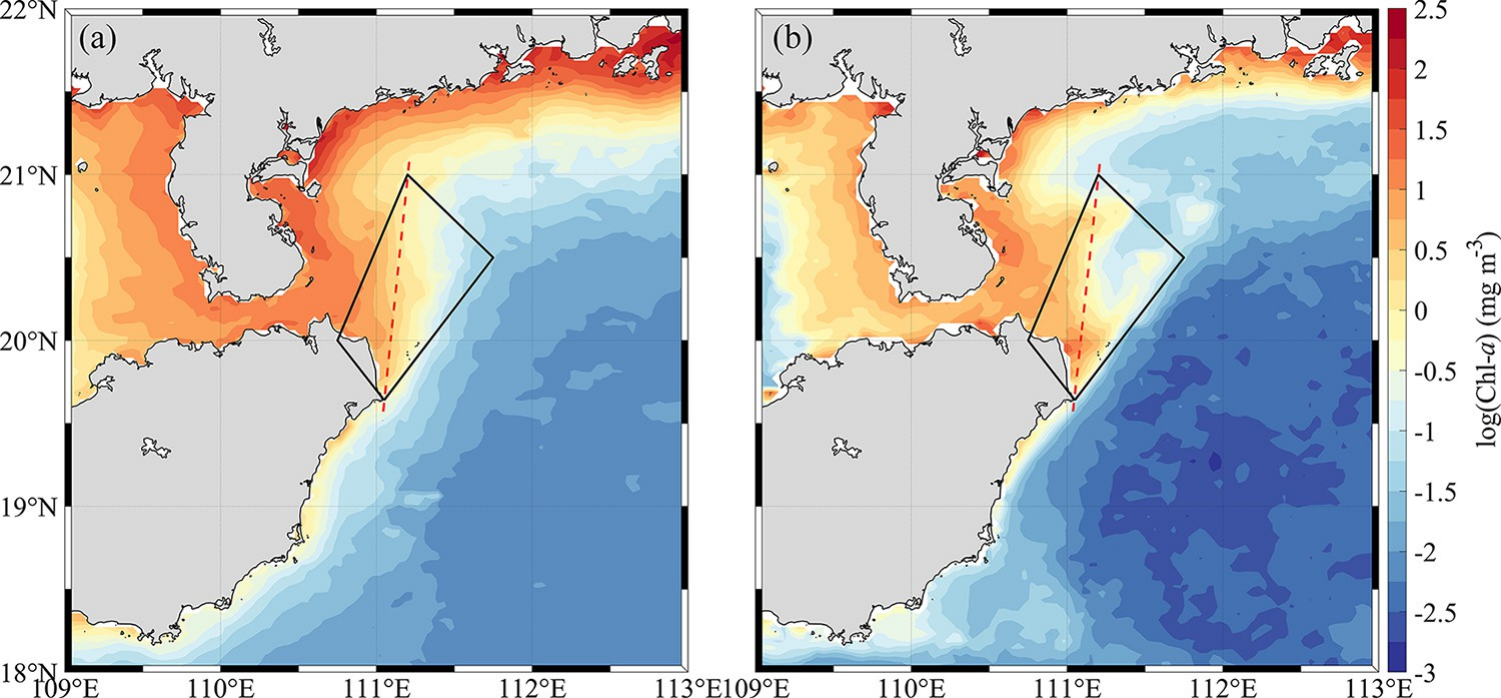
Distribution of Chl-*a* concentration in July. Monthly climatology of (a) chlorophyll-*a* (Chl-*a*) concentration averaged in July from 1998 to 2019 and (b) the summer (July) mean Chl-*a* concentration in 2020. The red dotted lines are the left and right partition lines of the HNEU area. The high-precision shoreline base-map data used in the map is derived from Global Self-consistent, Hierarchical, High-resolution Geography Database (GSHHG), which is the U.S. Government material and is not subject to copyright protection within the United States.

The Chl-*a* concentration is largely decreasing across the shoreline from nearshore to offshore (Fig 3). The area with high Chl-*a* concentration in the trapezoid is roughly consistent with the corresponding low temperature center in Fig 2, but the average Chl-*a* concentration of HNEU in 2020 is lower (Fig 3B). The spatial distribution of climatological Chl-*a* and Chl-*a* in 2020 exhibit also significant differences (Fig 3A and 3B). The HNEU is divided into two parts by the meridional diagonal of the trapezoid (red dashed line) with the high values of Chl-*a* concentration in the left part of both climatology and 2020. Compared with that of the climatology, the patch with the high Chl-*a* in the right part of the study region in 2020 is less.

### Relationship between CUI and Chl-*a* concentration

**Fig 4:**
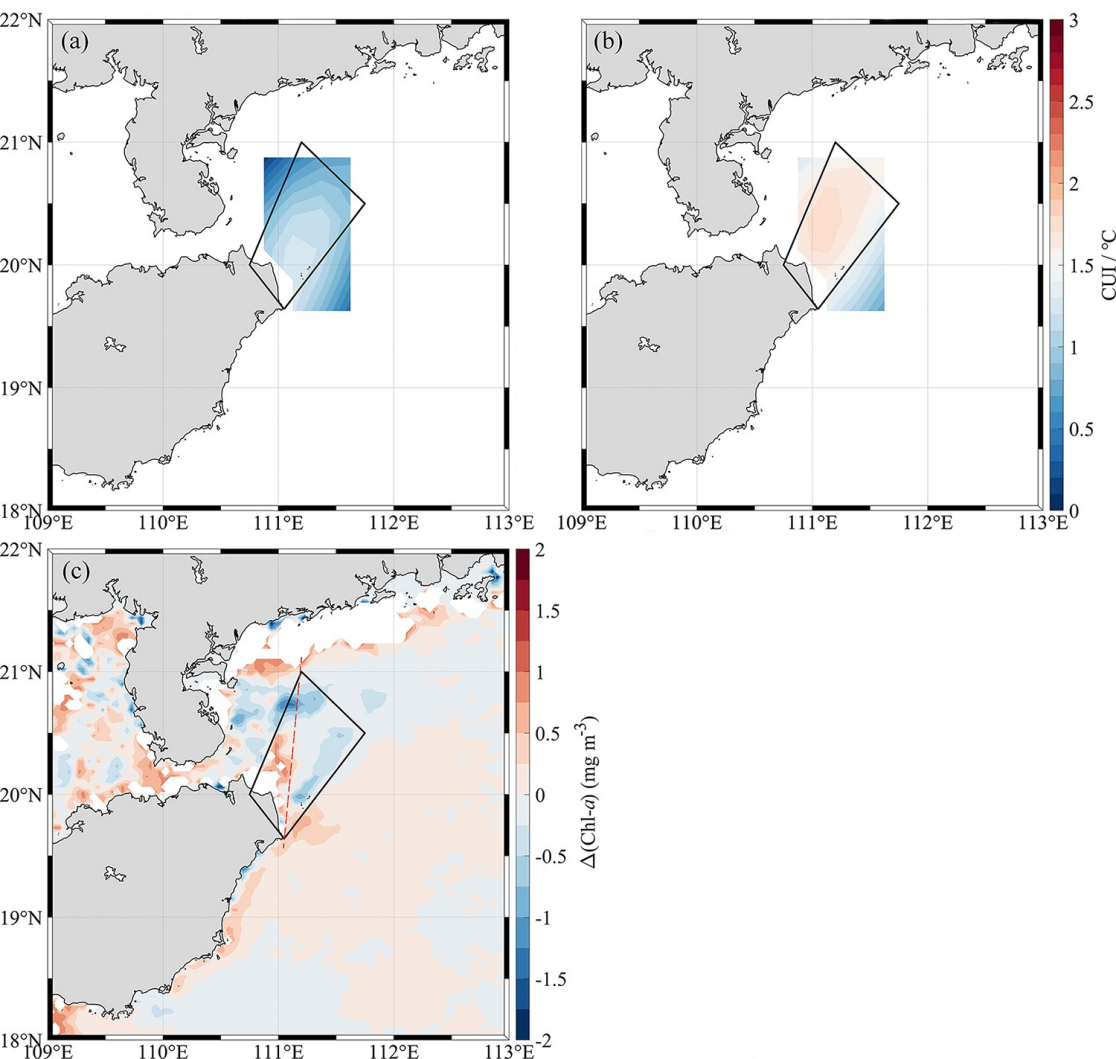
Upwelling intensity comparison and Chl-*a* concentration difference plots. Monthly average Coastal Upwelling Index (CUI) in (a) 2019 and (b) 2020, and (c) monthly average Chl-*a* concentration difference between 2019 and 2020 (Chl-*a* concentrations in 2019 minus Chl-*a* concentration in 2020) in the HNEU area in summer. The high-precision shoreline base-map data used in the map is derived from Global Self-consistent, Hierarchical, High-resolution Geography Database (GSHHG), which is the U.S. Government material and is not subject to copyright protection within the United States.

In order to explore the impact of typhoon on the distribution of phytoplankton in HNEU area in detail, we will focus on the comparison of individual cases. Considering that both 2019 and 2020 are El Nino years, and the background temperature field is roughly similar [33, 34]. We will take the summer of 2019 as a case study and compare it with 2020. Fig 4 shows a plot of the intensity of upwelling, that is, CUI in 2019 and 2020 (Fig 4A and 4B) and the difference in Chl-*a* concentration between 2019 and 2020 (Fig 4C) in the study area. In comparison, the average intensity of upwelling in July 2020 was significantly higher than that in July 2019 by ~1°C. But the mean Chl-*a* concentration in July 2020 (~ 0.92 mg m^-3^) was slightly lower than that in July 2019 (~ 1.11 mg m^-3^). In Fig 4C, the Chl-*a* concentration in most areas on the left side of the trapezoid in 2019 is about 0.5 mg m^-3^ higher than that in 2020. While the Chl-*a* concentration in the upper left part of the trapezoid in 2020 is significantly higher than that in 2019. It coincides with the position of the new upwelling center after moving northward (Fig 2B). Besides, the Chl-*a* concentration in the right half of the trapezoid located in Fig 4C is slightly higher in 2020.

**Fig 5:**
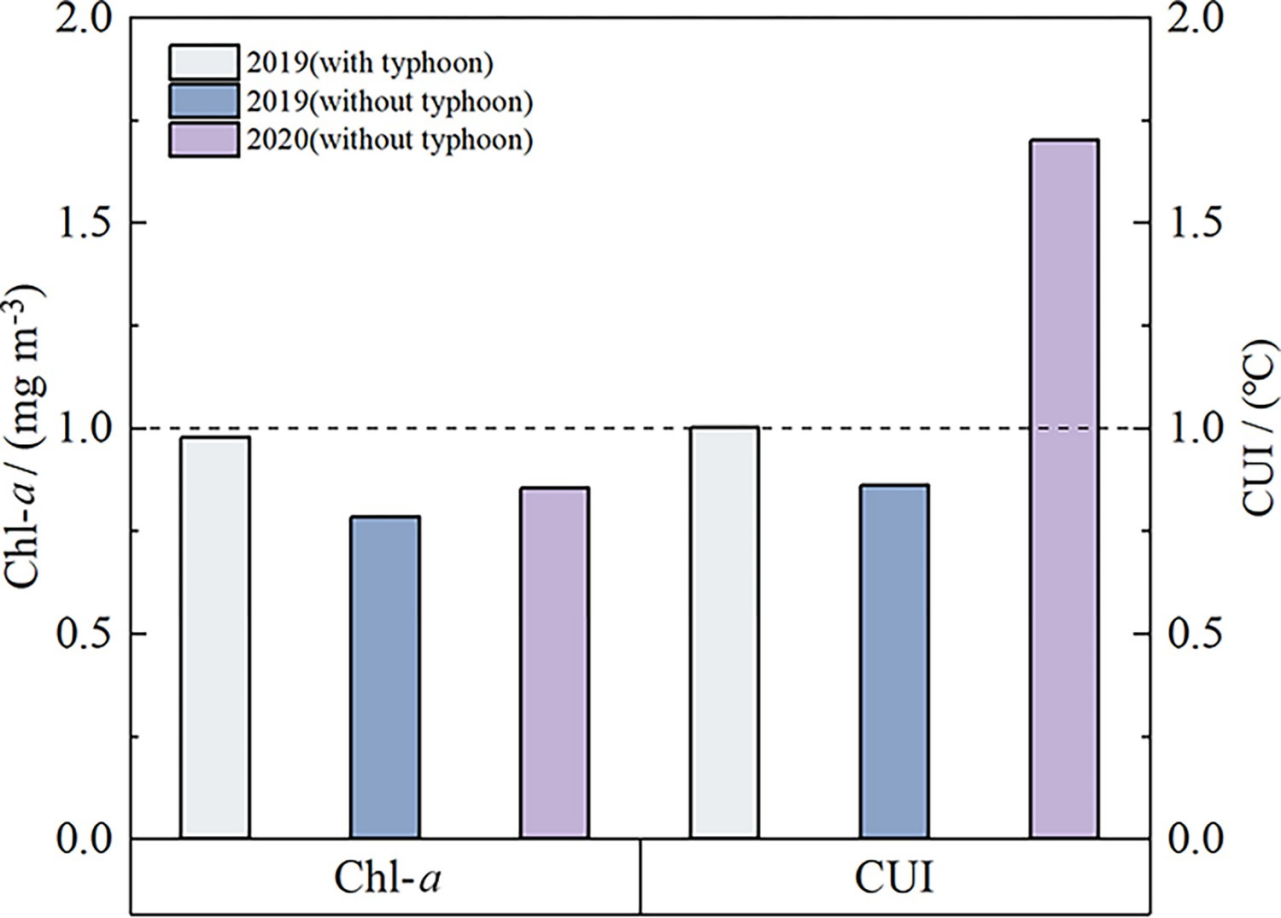
Comparison of CUI and Chl-*a* concentration. Comparative histogram of weekly average CUI and Chl-*a* concentration. Light blue columns are during typhoon crossing and one week after typhoon crossing in the summer of 2019. Dark blue columns are during the second to fourth week after typhoon crossing in the summer of 2019. Purple columns are 2020 corresponding to 2019 time period.

Further reduce the time scale to the typhoon-influenced period. We selected the CUI and Chl-*a* concentrations during the typhoon MUN (Table 1) impact period (during typhoon crossing and one week after typhoon crossing) in July 2019, the typhoon-free periods (second to fourth week after typhoon crossing) and the same period in 2020 for comparison. Fig 5 shows the comparison of Chl-*a* and CUI during the typhoon-influenced period and the typhoon-free period for these two years. During the typhoon-influenced period in 2019, the CUI in HNEU was slightly higher than that during the typhoon-free period in the same year. But the Chl-*a* concentration during typhoon crossing in 2019 is about 0.3 mg m^-3^ higher than that during the typhoon-free period in the same year. During the typhoon-free period in both 2019 and 2020, the CUI in 2020 is much higher than that in 2019, and the Chl-*a* concentration in 2020 (~ 0.80 mg m^-3^) is also significantly higher than that in 2019 (0.70 mg m^-3^). It is worth noting that the results show an interesting phenomenon: the CUI in 2020 was higher than that during typhoon crossing in 2019, but the Chl-*a* concentration shows the opposite trend.

**Table 1:** Specific information of MUN in 2019.

Number	Typhoon Naming	Typhoon generation			Intensity extremes	
		Time	Longitude	Latitude	Air pressure	Wind speed (m s^-1^)
1904	MUN	2019.07.02 21:00–07.04 14:00	111.0°E	18.8°N	992	18

**Fig 6:**
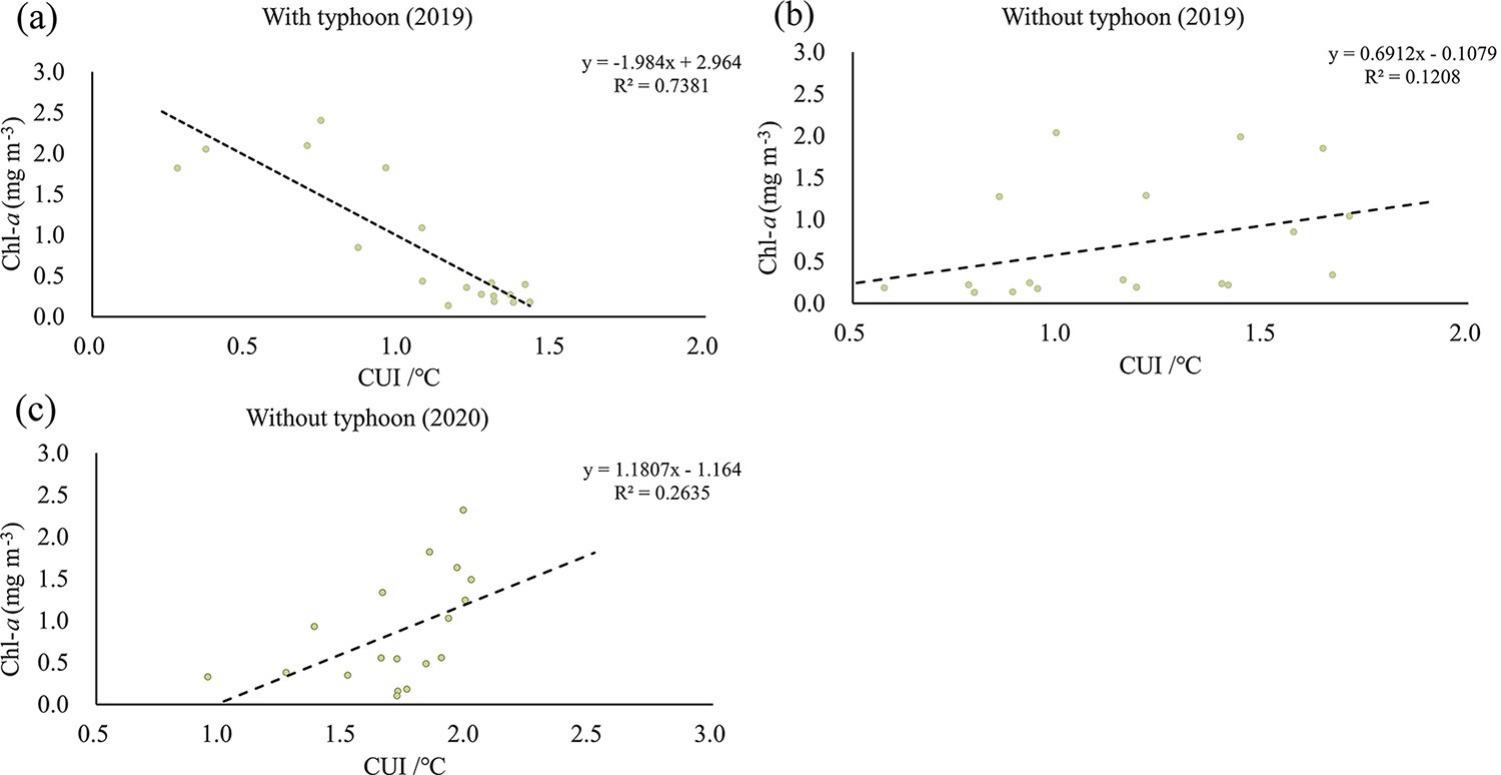
Correlation coefficients between CUI and Chl-*a* concentration. (a) For the typhoon-influenced period in 2019. (b) For the typhoon-free time period in 2019, and (c) for the corresponding time period in 2020. The spatial average data from 3 periods was used here, based on an area of (0.04° × 0.04°) in the study region.

The CUI is highly negatively correlated with Chl-*a* during the typhoon-influenced period in 2019 (R^2^ = 0.7381) (Fig 6). Typhoon crossing increases the vertical mixing of water bodies, which enhances upwelling and keeps nutrients in water bodies at a high level. But Chl-*a* concentration was still low at this time (below the trend line in Fig 6A). The stirring of the water column by the typhoon weakened in the later period, which made the upwelling gradually return to normal level. That is, with the weakening of CUI, phytoplankton will continue to use nutrients increased during typhoon crossing. This causes the concentration of Chl-*a* increased at first and then decreased. The CUI is moderately positively correlated with Chl-*a* during the typhoon-free period in 2019 (R^2^ = 0.1208). The CUI has a strong positive correlation with Chl-*a* during the typhoon-free period in 2020 (R^2^ = 0.2635). In these two typhoon-free periods, the Chl-*a* concentration all increased with the increase of CUI. From the above correlation results, it can be seen that there is a direct relationship between Chl-*a* and upwelling, which will be changed by typhoon crossing.

## Discussion

### The role of upwelling

The results about Chl-*a* and CUI suggested the upwelling may control the distribution of phytoplankton Chl-*a* in the HNEU under the typhoon-free influence in July of both 2019 and 2020. In the typhoon-free period, Chl-*a* concentration become higher with increasing upwelling strength. The strong correlation (Fig 6C) between CUI and Chl-*a* in July of 2020 implied also that upwelling may play important role in the high Chl-*a* concentration during the typhoon-free period. The HNEU occurs frequently in summer every year in the nearshore areas of Hainan Island. On the one hand, the nearshore wind stresses parallel to the coastlines can drive offshore horizontal Ekman transport during southwest monsoon, triggering coastal upwelling [6]. On the other side, Ekman pumping induced by wind stress curls has also a significant effect in the growth of phytoplankton in the upwelling area [35]. Its contribution to upwelling may be greater than that of coastal wind stress in our study region [4]. The Ekman pumping transported the low-temperature and nutrient-rich seawater from the bottom layer to the surface layer to replenish the missing water, leading to sufficient nutrients in surface water in HNEU area and suitable conditions of phytoplankton growth. In turn, it led also to a significant increase in Chl-*a* concentration (Fig 3B). Moreover, the local WS is high during the typhoon-free period (Fig 2C and 2D). The CUI caused by the corresponding coastal wind increases, so does the Chl-*a* concentration (Fig 4). And the southeasterly wind is favorable for the formation of upwelling in the northeast of Hainan Island [2]. When under the action of stronger southeasterly winds, the upwelling will be pushed northward to the sea area closer to the Leizhou Peninsula. The northward-moving upwelling can also carry high Chl-*a* concentration water, producing a plume of high Chl-*a* concentration during the typhoon-free period in the upper left part of the HNEU (Fig 4C). There was stronger CUI in 2020 than in 2019 during typhoon-free periods., which is possibly the reason that higher the Chl-*a* concentration in the HNEU area in the summer in 2020 than in 2019.

### The role of typhoons

As an extreme weather event, typhoons will have a significant impact on upper oceans in one short time [23]. The response of seawater to typhoons could alter nutrient availability, the activity of phytoplankton, and impact the concentration of Chl-*a* in the euphotic zone. The occurrence of typhoons often causes eddies and strong winds, which can alter the original flow field of the ocean, induce mixing, affect hydrography and nutrient transport, and lead to changes in the biomass and community structure of phytoplankton [36–38]. In the non-upwelling area, the increase of Chl-*a* concentration was frequently caused by typhoon-enhanced eddy pumping nutrients into the surface layer [39]. This phenomenon is an important process in the biogeochemical cycle [39, 40]. Therefore, when the typhoon is superimposed with the original upwelling system, it will probably have a more prominent impact on the growth of phytoplankton in that area.

**Fig 7:**
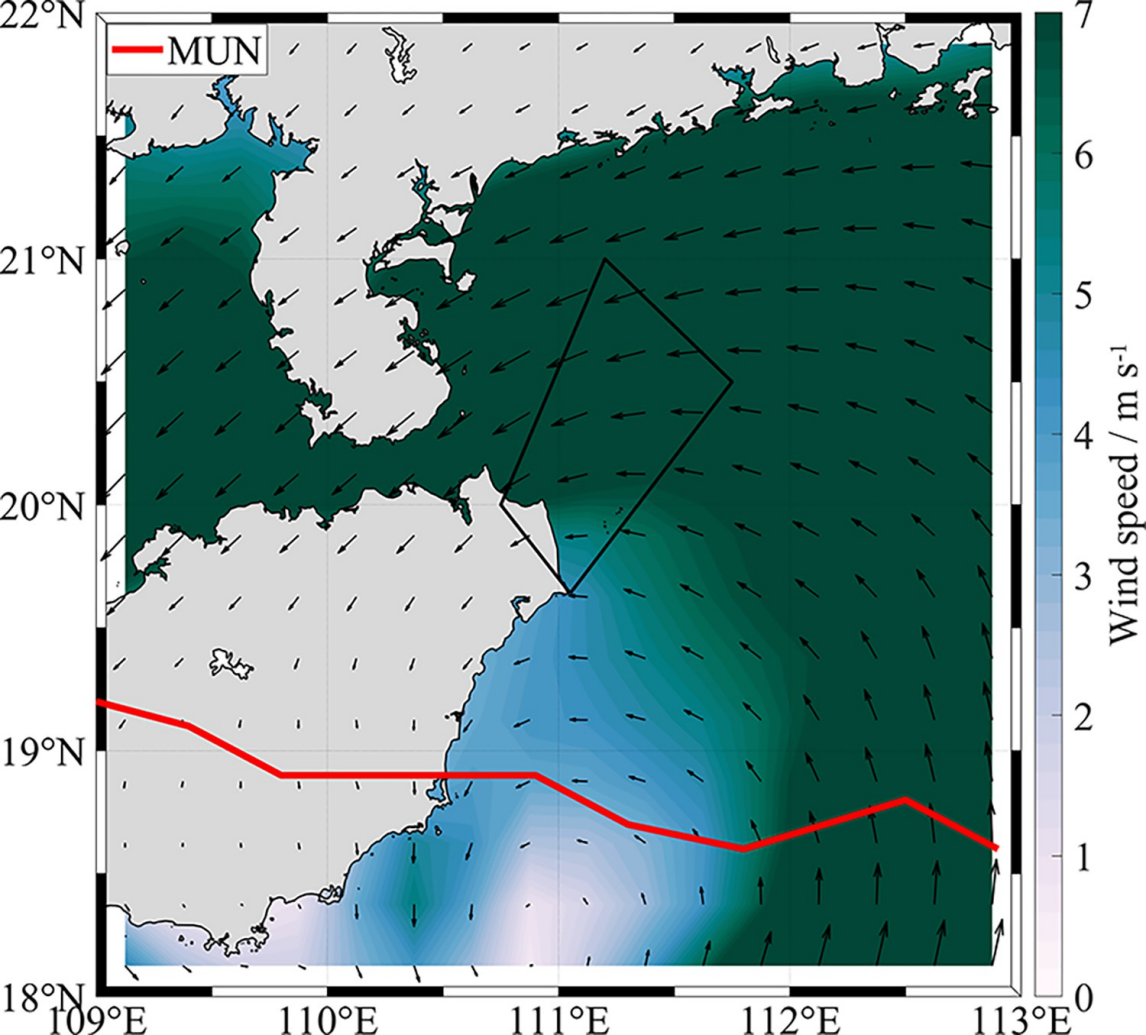
WS on the day of typhoon crossing. Red line is path of typhoon MUN in the study area in the summer of 2019. The high-precision shoreline base-map data used in the map is derived from Global Self-consistent, Hierarchical, High-resolution Geography Database (GSHHG), which is the U.S. Government material and is not subject to copyright protection within the United States.

According to our study results, the typhoon passage in the summer of 2019 enhanced evidently influence of upwelling on Chl-*a* concentration. When MUN (Table 1) passes on the south side of HNEU, the original southwest wind is changed into northeasterly wind (Fig 7). It will change the original upwelling system. Due to the shallow water depth in the area (Fig 1B), strong typhoons can cause rapid and violent mixing of surface waters. The vertical mixing of water accelerates the upward motion of the bottom cold water in the short term [17], resulting in the enhancement of CUI (Fig 5). Typhoons passages can bring rainfall, causing a surge of runoff in the surrounding land source areas [41, 42]. The concentration of granular phosphorus and granular silicon in the water increased significantly [43]. The large input of nutrients in the short term would allow the present abundant phytoplankton in the HNEU area to receive more adequate nutrients. After typhoon-influenced period, the wind speed gradually decreases to normal speed and the offshore Ekman transport in the surface layer drops back [44]. At the same time, the onshore current along the slope also decreases accordingly. Thus, the surface waters to the right of the typhoon path appear to warm up (i.e., the strength of the upwelling returns generally to pre-typhoon levels) [45, 46]. At this moment, the water is in a eutrophic state which can support more phytoplankton growth, leading to higher Chl-*a* concentration about one week after the passage, due to lagging response of phytoplankton to nutrients [47].

We also found an interesting point: even though the CUI in 2020 was much higher than the CUI during typhoon-influenced period in 2019, the Chl-*a* concentration was still lower than in 2019. It can be inferred that the mixing effect induced by the strong wind speed of typhoons also brings subsurface nutrients to the surface layer. Additionally, it lifts the subsurface Chl-*a* maximum layer to the surface layer. This results in a rapid increase of Chl-*a* in the surface layer in a short time, which is stronger than the effect of upwelling alone on phytoplankton [48]. Therefore, although the typhoon has a limited enhancement of upwelling, the Chl-*a* concentration of HNEU still increases more than that during typhoon-free period, due to the superposition of the additional effects including stronger water mixing and heavier rainfall and land-runoff during typhoon crossing.

## Conclusion

**Fig 8:**
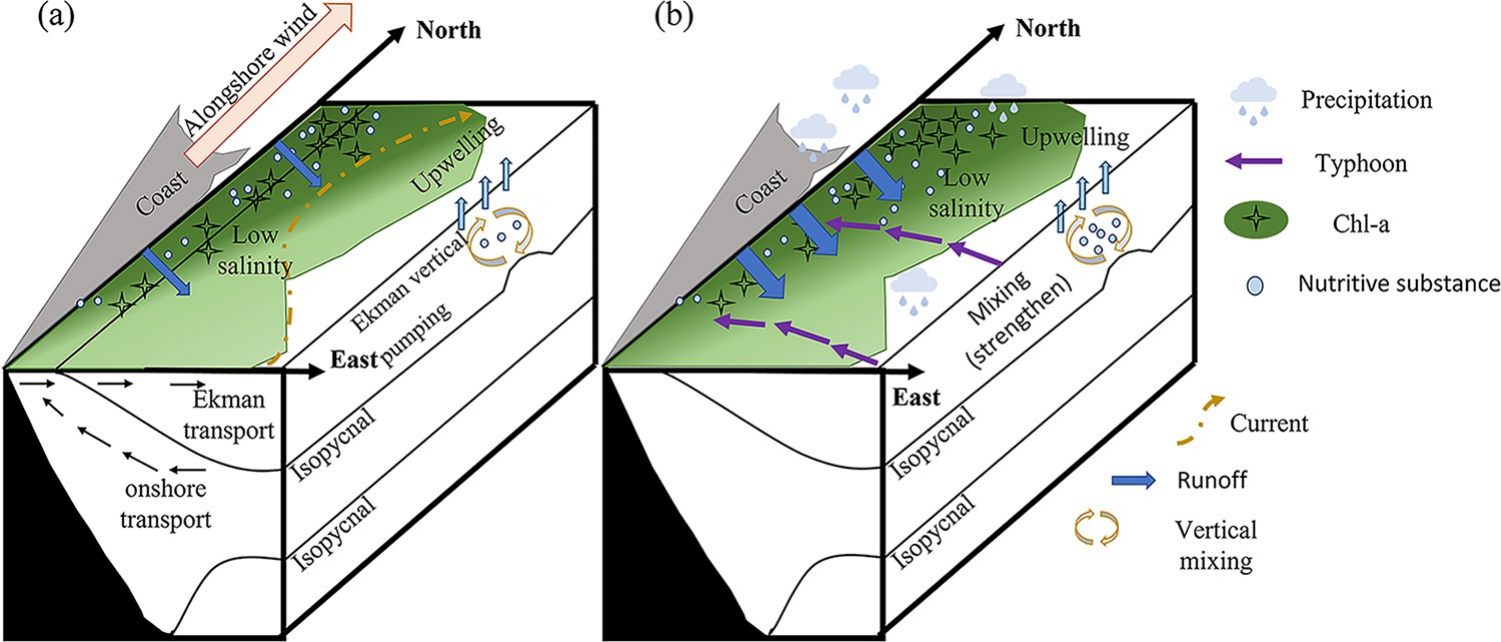
Map of possible mechanisms affecting the ecological environment of the HNEU area. (a) During the typhoon-influenced period and (b) during typhoon-free period.

This study focuses on the effects of typhoon or without typhoon on the ecological environment of the HNEU area. Large differences in the response of phytoplankton Chl-*a* to typhoon crossing (2019) or without typhoon (i.e. 2020) were observed in HNEU area during summer. Though the CUI of HNEU area is significantly higher than the typhoon-influenced period for many years, the phytoplankton Chl-*a* concentration is lower, suggesting typhoon would exert an important factor for the above phenomena. Here, a schematic diagram of the ecological processes acting on the HNEU area with and without typhoon is presented (Fig 8): Upwelling dominates the distribution of phytoplankton Chl-*a* concentration in HNEU area during the typhoon-free period (i.e. higher Chl-a with high CUI). Normally, Ekman pumping caused by wind stress curl plays a more important role in upwelling than Ekman transport by wind stresses parallel to the coastline. Response of phytoplankton Chl-*a* concentration to typhoons are more evident than that upwelling in HNEU area. The superposition effect of stronger sea water mixing by strong wind stress during typhoon and enhanced upwelling increases further the vertical nutrient transport (Fig 8B). Although the typhoon has a limited enhancement of upwelling, the more evident increase in Chl-*a* concentration with typhoon can attribute to the superposition of the additional effects including stronger water mixing and heavier rainfall and land-runoff during typhoon crossing.

## Supporting information

S1 Data (ZIP)
